# Determining the origin of synchronous multifocal bladder cancer by exome sequencing

**DOI:** 10.1186/s12885-015-1859-8

**Published:** 2015-11-09

**Authors:** Ömer Acar, Ezgi Özkurt, Gulfem Demir, Hilal Saraç, Can Alkan, Tarık Esen, Mehmet Somel, Nathan A. Lack

**Affiliations:** 1 Department of Urology, School of Medicine, Koc University, Istanbul, Turkey; 2 Department of Urology, VKF American Hospital, Istanbul, Turkey; 3 Department of Biological Sciences, Middle East Technical University, Ankara, Turkey; 4 Department of Computer Engineering, Bilkent University, Ankara, Turkey; 5 School of Medicine, Koc University, Istanbul, Turkey

**Keywords:** Multifocal bladder cancer, Next-generation sequencing, Population genetics, APOBEC deaminase

## Abstract

**Background:**

Synchronous multifocal tumours are commonly observed in urothelial carcinomas of the bladder. The origin of these physically independent tumours has been proposed to occur by either intraluminal migration (clonal) or spontaneous transformation of multiple cells by carcinogens (field effect). It is unclear which model is correct, with several studies supporting both hypotheses. A potential cause of this uncertainty may be the small number of genetic mutations previously used to quantify the relationship between these tumours.

**Methods:**

To better understand the genetic lineage of these tumours we conducted exome sequencing of synchronous multifocal pTa urothelial bladder cancers at a high depth, using multiple samples from three patients.

**Results:**

Phylogenetic analysis of high confidence single nucleotide variants (SNV) demonstrated that the sequenced multifocal bladder cancers arose from a clonal origin in all three patients (bootstrap value 100 %). Interestingly, in two patients the most common type of tumour-associated SNVs were cytosine mutations of TpC* dinucleotides (Fisher’s exact test *p* < 10^−41^), likely caused by APOBEC-mediated deamination. Incorporating these results into our clonal model, we found that TpC* type mutations occurred 2-5× more often among SNVs on the ancestral branches than in the more recent private branches (*p* < 10^−4^) suggesting that TpC* mutations largely occurred early in the development of the tumour.

**Conclusions:**

These results demonstrate that synchronous multifocal bladder cancers frequently arise from a clonal origin. Our data also suggests that APOBEC-mediated mutations occur early in the development of the tumour and may be a driver of tumourigenesis in non-muscle invasive urothelial bladder cancer.

**Electronic supplementary material:**

The online version of this article (doi:10.1186/s12885-015-1859-8) contains supplementary material, which is available to authorized users.

## Background

**Fig. 1:**
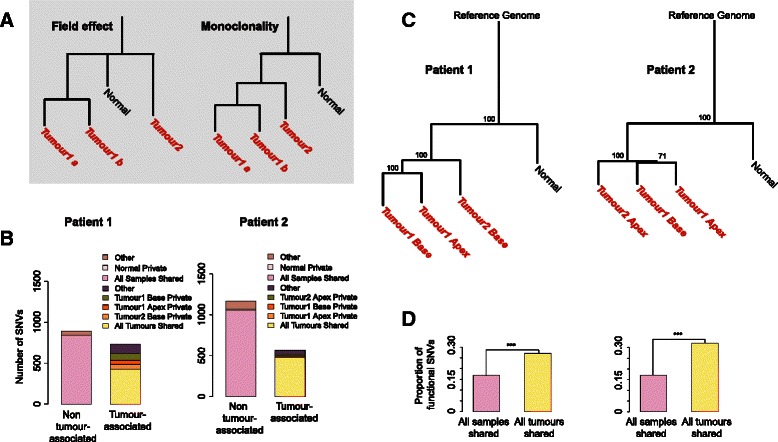
SNV distribution and phylogeny of tumours. **a** Hypothetical phylogenies with the field effect and monoclonal origin hypotheses. **b** Number of SNVs with respect to occurrence among samples. Tumour 1 Apex, Tumour 1 Base, Tumour 2 Base/Apex, Normal private: SNVs *only* in that sample. As there are only 3 normal private mutations their bar is too small to be visually noticeable. All samples shared: SNVs in all 4 samples, representing the individual genotype. Other: SNVs in the normal mucosa sample *and* in one or in two tumour samples. All tumours shared: SNVs in all 3 tumour samples but not in normal mucosa. Other tumour-associated: SNVs in found in one or two but not all tumours and not in normal mucosa. **c** Neighbour-joining tree of the 4 samples and the human reference genome based on 1628–1733 high-confidence SNVs. Bootstrap support for each internal node is indicated. **d** Proportion of functional SNVs among all SNVs shared among all 4 samples (*n* = 143–182), and SNVs shared among all 3 tumours (*n* = 116–152). ***: Fisher’s exact test *p* < 0.001

Synchronous multifocal tumours are present in ~30 % of all non-muscle invasive urothelial carcinomas of the bladder. Two competing theories have been proposed to describe how these physically separated independent tumours arise. The clonal hypothesis suggests that multifocal tumours are formed by intraluminal or intraepithelial migration of cells that are shed from a founder tumour. In contrast, the field hypothesis proposes that a large area of cells is first partially transformed by a carcinogen and then subsequently acquires additional mutations that induce neoplastic transformation. As cancer occurs by the sequential accumulation of transformative mutations, each model would produce a different genetic signature (Fig. 1a). Various studies investigating the origin of multifocal bladder cancer have produced conflicting reports. Classic work characterizing the X-chromosomal inactivation patterns in female patients [1] and microsatellite loss of heterozygosity (LOH) [2] clearly demonstrated a clonal origin. Further, tumours analysed by array-based comparative genomic hybridization also showed monoclonality [3]. However, in a study of 21 bladder cancer patients, ~30 % of patients demonstrated significant LOH allelic differences among tumours with >40 % of patients showing some allelic variance, suggesting that these tumours arose from a field effect [4]. Additional research with microdissected bladder tumour samples also demonstrated similar results in accordance with a field effect hypothesis [5, 6]. Interestingly, in a recent study of metachronous multifocal tumours, the authors clearly demonstrated that these arose from a clonal origin [7].

A potential cause of this variability could be attributed to the small number of genetic mutations used to quantify the relationship of the tumours. Given the intrinsic genetic instability of cancer, the mutational markers selected to quantify the relationship between these tumours could drastically alter their predicted molecular origins. We therefore hypothesized that the thousands of unbiased genetic markers generated through next-generation sequencing would be ideally suited to determine the origin of synchronous multifocal bladder cancer.

## Methods

### Ethics and consent

Ethical approval was obtained for this study from the Koç University Institutional Review Board (2012.017.IRB2.007) in accordance with the Declaration of Helsinki. Written consent to participate in this study was obtained from all patients.

### Clinical sample collection

Synchronous multifocal tumours were obtained from male patients (62–72 years old) with pTA papillary urothelial carcinoma. During transurethral resection, all macroscopically visible tumours were resected and a sample from the normal mucosa was taken for study purposes. Normal mucosa sample was located equidistantly with respect to the tumoural foci. As a result, a total of four samples (three neoplastic, one normal) were collected from each patient.

Samples were washed in saline solution and then snap frozen in liquid nitrogen within 10 min of the resection.

### Molecular biology methodology

Genomic DNA was isolated from samples following mechanical shearing using the Qiagen QIAmp DNA kit as per the manufacturer instructions. Illumina adaptor sequences were added to sheared and blunted gDNA by TA ligation. Exomic sequences were captured with Agilent SureSelect v5 and sequenced on an Illumina HiSeq 2000 with 100bp paired end reads at 100× coverage by Centrillion BioScience as a contract service.

### Sequence alignment

We aligned the resulting sequencing data (Additional file 1: Table S1) to the reference human genome (hg19/NCBI GRCh37) using the BWA aligner (version 0.7.10) using the “mem” algorithm with default options and paired-end mode [8]. Following this, to correct for mapping biases, we used the GATK tool for realigning indel-containing reads [9]. GATK UnifiedGenotyper in multi-sample mode was used to generate SNV and indel callsets by pooling read data from the four samples of a patient. The Variant Quality Score Recalibration filter was used from the GATK resource bundle version 2.5 to help minimize false positives. Since this project is focused on cancer- and tissue- specific variants, we removed any SNV or indel prediction that is represented in dbSNP version 138. Therefore, common SNPs were not included in the genetic analysis. In our final SNV dataset we only included SNVs and indels where the genotype was reliably detected (passed the GATK quality filter) and was supported by ≥4 reads in all four samples. This procedure was repeated independently for each patient.

### Population genetics analysis

Downstream bioinformatic analysis was conducted using R and Python programming languages. The SNV data was converted into binary form, with heterozygous or homozygous non-reference alleles called “1”, and homozygous reference alleles called “0”. The same approach was applied to the indel data. We added the reference genome (“0” s at all positions) to these datasets. The binary datasets were used to calculate Euclidean distance matrices among samples using the R “dist” function. We then constructed phylogenetic trees using the R “ape” package’s [10] “bionj” algorithm – a variant of the neighbor-joining algorithm suggested to perform well when branch lengths are heterogeneous, as in our case [11]. The “reference genome” was used to root the tree. Using the more simple Unweighted Pair Group Method with Arithmetic Mean algorithm produced qualitatively identical results (data not shown). We performed 10,000 bootstraps using the “boots.phylo” function of the R “ape” package.

### Analysis of indels

We also identified 2130–2555 indels in the three patients (Additional file 1: Table S1) using the GATK analysis procedure described above. We repeated the same phylogenetic analysis as above using indels, but the phylogenetic relationships could not be resolved (low bootstrap support) (Additional file 2: Figure S1). Observing a strong phylogenetic signal using SNVs, but not using indels can be explained by homoplasies (convergent evolution), but more likely, high technical noise in indel calls in exome sequencing at this coverage. We did not include indels in further analysis.

### Functional analysis

We used the “snpEff” software [12] to annotate SNVs and indels according to their impact on protein structure. All mutations predicted to have low, moderate or high impact on protein sequence (e.g. loss of splice sites, non-synonymous substitutions, stop-codon insertions) were considered “functional.” We compared ratios of functional vs. non-functional mutations between SNV sets (e.g. those shared among all samples vs. those shared only among the 3 tumour samples) with the Fisher’s exact test using the R “fisher.test” function.

### Mutation type analysis

We classified SNVs based on the dinucleotide sequence context following Lawrence et al. [13], where we recorded the nucleotide preceding an SNV using the human reference genome (hg19). To simplify the analysis, following Nordentoft et al. [7] we only considered A and C positions in the reference genome and the preceding nucleotide, on each strand, and made a list of all SNVs in dinucleotide context. We compared the frequencies of TpC* vs. non-TpC* mutations between SNV sets (e.g. SNVs shared among all samples, SNVs shared only among the 3 tumour samples, and SNVs private to tumours) with the Fisher’s exact test. We similarly compared dinucleotide frequencies taking into account the resulting mutation, as well as trinucleotide frequencies (Additional file 3: Table S3).

### Candidate driver gene analysis

We identified a series of potential driver mutations from those genes frequently mutated in bladder cancer in the COSMIC [14] and ATLAS [15] databases (as of October 1^st^, 2014). This yielded 94 candidate driver mutations. We then checked for overlap between this list and genes containing SNVs that are both functional (based on the “snpEff” software; see above) and are mutated in at least one tumour sample.

## Results

### Exome sequencing of synchronous multifocal tumours

Synchronous multifocal tumour samples were surgically removed from the bladder during transurethral resection. Ethical approval and patient consent were obtained for all sample collections. Material from patients was only sampled if the tumours were physically separated (>1cm). Resected material from three patients with a low/high grade, pTa, urothelial carcinoma with very little stromal cell contamination was used for further analysis. In each patient, genomic DNA was isolated from the base and apex of a single tumour, the base of a second tumour and the normal bladder mucosa (four samples/patient). Multiple samples were taken from a single tumour to determine the intratumoural mutation heterogeneity. The exome regions of gDNA were enriched and sequenced with paired-end Illumina sequencing at 100× coverage. High-depth coverage was used to increase confidence in mutation calling. The resulting sequences (51–66 million reads/sample) were aligned to the human reference genome (GRCH37) with 94 % of the reads successfully mapping (Additional file 1: Table S1). By using strict filtering criteria (≥4 reads in each sample, see Material and Methods), between 1628–1733 single nucleotide variants (SNV) were identified in each patient.

In the first two patients, approximately half (52–61 %) of these SNVs were shared among all samples including the normal mucosa. These SNVs were assumed to represent the individual’s unique genotype, i.e. germline variants. Almost all of the remaining SNVs (33–45 %) were observed in at least one tumour sample but not in the normal mucosa. These somatic mutations were therefore defined as “tumour-associated SNVs” (*n* = 564–734). In these patients, the majority of the tumour-associated SNVs were shared among all three tumour samples (58–84 %), with only 0.6–5 % (*n* = 11–80 SNVs) private to each tumour (Fig. 1b). In contrast, in the third patient, 80 % of SNVs were shared among all samples, while only 15 % of the SNVs were tumour-associated, and only 2 % of these were shared among all tumour samples (Additional file 4: Figure S2). Therefore, in the third patient, the normal sample’s genetic profile appears very similar to those of the tumour samples.

We then built phylogenetic trees based on the SNVs detected in each patient, and bootstrapped over these mutations. The trees for the first two patients clearly demonstrated that the tumour samples are monophyletic (of clonal origin) with significantly similar genetic signatures to each other compared to the normal tissue (Fig. 1c, bootstrap value 100 %). Notably, the bootstrap test ensures that the phylogenetic trees are robust to any random sequencing errors. In both trees, the tumour branches were markedly longer than the normal mucosa branch, indicating an excess of mutation accumulation in the tumour lineages. In contrast, the “normal” sample clustered together with the other tumour samples with high confidence in the third patient’s phylogenetic tree (Additional file 5: Figure S3). In this patient, neither tumour branch was longer than the “normal” sample branch, in contrast to what was observed in the other two patients. We therefore concluded that the “normal” sample from the third patient contained contaminating neoplastic material. Supporting this theory, the “normal” sample was found to have functional mutations in many classical bladder cancer tumour suppressors (Additional file 6: Table S2). Meanwhile, we noted that this patient’s first tumour’s apex and second tumour’s base clustered together (Additional file 5: Figure S3), to the exclusion of the first tumour’s base. The second tumour is therefore a phylogenetic sub-branch of the first tumour. This can only be explained by a monoclonal origin of the two tumours, rather than the field effect hypothesis. Together, these results strongly support a clonal origin for all of the collected synchronous multifocal tumours.

We then investigated the functionality of tumour-associated SNVs in the first two patients, relative to SNVs representing the individual’s genotype. Classifying all 1628–1733 SNVs according to their impact on protein sequence revealed that 350–354 (20–22 %) of these mutations were potentially functional, i.e. altered protein sequence or splicing pattern. Such functional mutations were about 1.8–2.3 times more common among SNVs shared among tumours than those representing the individual’s genotype (Fig. 1d, Fisher’s exact test *p* < 10^−5^).

### APOBEC mutations in multifocal tumours

**Fig. 2:**
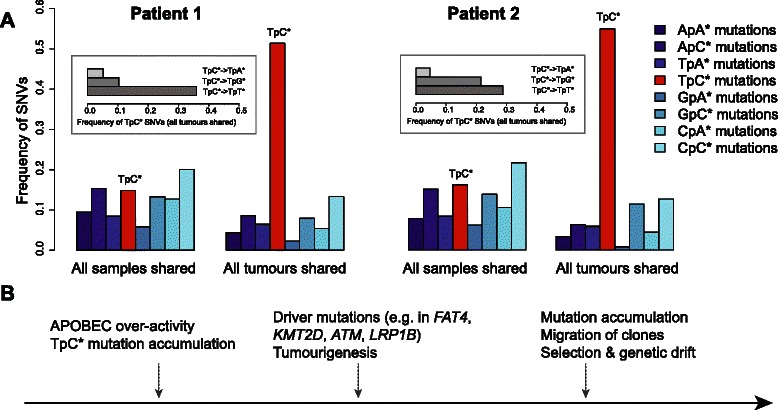
**a** SNV frequencies in dinucleotide context. Frequencies are compared between SNVs among all SNVs shared among all 4 samples (*n* = 842–1059), and SNVs shared among all 3 tumours (*n* = 428–473). Asterisks indicate which base is mutated; e.g. TpC* stands for TpC- > TpA, TpC- > TpG, or TpC- > TpT. Inset shows the frequency of substitutions in all tumours at TpC* sites. We only consider A or C mutations in either strand and their 5’ nucleotides, following [9]. **b** Proposed development of the bladder cancer lesion from initiation of transformation to migration of the clonal tumours

A recent study observed that TpC* mutations (where a C preceded by a T is mutated) occur at a higher frequency in bladder, cervical and head-and-neck cancers [16]. This pattern was proposed to be caused by over-activation of APOBEC family single stranded RNA/DNA editing enzymes; APOBECs can cause widespread cytosine deamination, possibly in response to retroviral infection [17]. We therefore asked whether TpC* substitutions might also accumulate at high rate in the tumours sampled. Indeed, we found higher TpC* substitution frequencies, particularly TpC*- > TpT*/TpG*, relative to non-tumour associated mutations in two of first two patients (Fig. 2a, Additional file 3: Table S3 and Additional file 7: Table S4). Compared to the SNVs representing the individual’s genotype (*n* = 842–1059), the SNVs shared among all tumours (*n* = 428–473) had a 6–6.3 fold higher proportion of TpC* mutations (Fisher’s exact test *p* < 10^−41^). Furthermore, TpC* mutations shared among tumours were most frequent in the context of TpC*pA, consistent with the APOBEC3B mutational signature [18] (Additional file 3: Table S3). Given that these tumours arise from a common origin, we can roughly assess when these TpC* mutations occurred based on the distribution of this particular mutation within the phylogeny. With this model, we found that in both patients, TpC* mutations were 2.5–4.6 times more common along the ancestral tumour branch (SNVs shared among tumour samples, or “trunk” [19]) relative to more recent branches (SNVs private to each tumour) (*p*-value < 10^−4^, Additional file 8: Table S5). This data suggests that APOBEC-mediated mutations occurred early in the development of the tumours in these two patients. Supporting this model, we found that among missense SNVs in common bladder cancer “driver” genes, as defined by the COSMIC and ATLAS databases, approximately half were TpC* mutations (Additional file 6: Table S2). Meanwhile, in the third patient, TpC* mutation rate did not show a comparable elevation (Additional file 9: Figure S4) indicating that this mutation pattern is not ubiquitous for bladder cancer.

## Discussion

Previous studies characterizing the origins of synchronous multifocal bladder cancer produced conflicting results, with data supporting both a clonal and field effect. This potentially could be due to the limited number of genetic markers used in many of these previous studies. By analyzing a comprehensive set of markers with exome sequencing it is possible to overcome this limitation. Sequencing data also provides detailed information that can indicate the possible mechanisms behind each tumourigenesis event.

Therefore, in this study we conducted next-generation sequencing and identified thousands of unique SNVs per patient. With this data, we were able to utilize conventional population genetic models to determine the relationship between these multifocal tumours. Our data clearly demonstrated that these tumours arose from a clonal origin in all three patients sampled. While one of the patients appears to have a neoplastic contamination in its “normal” sample, this patient’s data is still consistent with the monoclonal origin model, as there is considerable overlap in the mutations found across the patient’s samples. Our finding parallels a recent report indicating a clonal origin for four metachronous tumour pairs that were characterized by genome sequencing [7]. Such similarity raises the interesting possibility that metachronous tumours may reflect synchronous tumours that have been “seeded” but not yet grown at the time of surgery. Both tumour types appear to arise from a clonal origin, suggesting a similar origin. However, further studies will be needed to characterize this potential relationship.

Once developed this clonal model can be used to provide insight into the evolutionary timing of the cancer. If a mutation happens very early in the development of the cancer it will be shared with all samples, however a mutation that occurs late will only be found in a single region or tumour. We applied this concept to study the timing of TpC* mutation accumulation. Recent work has demonstrated that this class of mutations occurs by over-activation of the cytosine deaminase APOBEC3B [16, 17]. This enzyme is believed to be important in the host-defence of retroviruses and transposons; however large-scale cancer genome sequencing studies have demonstrated that it is also a major mutagenic factor in many types of cancer [18]. Our results demonstrated that in two patients, APOBEC-mediated mutations occurred very early in tumour development and could potentially be important in the initial neoplastic transformation (Fig. 2b). This is seen in the high frequency of TpC* mutations in potential “driver” mutations, including missense TpC* mutations in well-known genes such as *KMTD2* and *ATM* shared across all tumours of a patient (Additional file 6: Table S2). Our result deviates from recent studies reporting accelerating of APOBEC-mediated mutations in lung cancer in smokers [19], or constitutive accumulation in early and late metachronous bladder cancer tumours [7]. This difference may reflect variation in the role of APOBEC activity in different cancers. Our finding also suggests that, as TpC* mutations primarily occur early in the development of the cancer, therapeutic targeting of APOBEC3B may not be suitable in non-muscle invasive bladder cancer.

Overall this study demonstrates the utility of exome sequencing and population genetic analysis in characterizing the molecular evolution of synchronous bladder cancer tumours.

## Conclusions

By using next-generation sequencing to identify a large number of unique mutations our study clearly demonstrates that multifocal tumours primarily arise from a clonal origin. Interestingly our work also suggests that APOBEC mutations occur relatively early in the development of non-muscle invasive bladder cancer.

## Additional files

Additional file 1: Table S1. Number of reads and number of detected SNVs and indels. Cov: coverage. The strict filtering criteria include the GATK quality score (“PASS”), excluding segmental duplications, removing SNVs included in dbSNPv.138, only including SNVs reliably genotyped in all 4 samples, and identified at least in 4 reads. See Material and Methods for details. (XLS 29 kb)
Additional file 2: Figure S1. Neighbour-joining tree of indels in the 4 samples and the human reference genome based on 2130–2555 indels. Bootstrap support for each internal node is indicated. (PDF 301 kb)
Additional file 3: Table S3. Odd’s ratio and Fisher’s exact test p-values for a frequency difference between SNVs shared among all tumours and SNVs shared among all samples, with respect to dinucleotide context and trinucleotide context. The table includes results for Patients 1 and 2. Bold lines show significant results with odd’s ratio > 1. (XLS 25 kb)
Additional file 4: Figure S2. Number of SNVs with respect to occurrence among samples of the Patient 3. Tumour 1 Apex, Tumour 1 Base, Tumour 2 Base, Normal private: SNVs *only* in that sample. All samples shared: SNVs in all 4 samples. Other: SNVs in the normal mucosa sample *and* in one or in two tumour samples. (PDF 140 kb)
Additional file 5: Figure S3. Neighbour-joining tree of the 4 samples from Patient 3 and the human reference genome based on 1628 high-confidence SNVs. (PDF 119 kb)
Additional file 6: Table S2. Potential driver mutations in samples identified from COSMIC or ATLAS databases and literature. (XLS 33 kb)
Additional file 7: Table S4. Odd’s ratio and Fisher’s exact test p-values for a frequency difference between SNVs shared among all tumours and SNVs shared among all samples, with respect to dinucleotide context and resulting mutations. The table includes results for Patients 1 and 2. Bold lines show significant results with odd’s ratio > 1. (XLS 24 kb)
Additional file 8: Table S5. Odd’s ratio and Fisher’s exact test p-values for a frequency difference between SNVs shared among all tumours and SNVs found private to tumours, with respect to dinucleotide context. The table includes results for Patients 1 and 2. Bold lines show significant results with odd’s ratio > 1. (XLS 20 kb)
Additional file 9: Figure S4. Dinucleotide specific SNV frequencies in Patient 3. Frequencies are compared between SNVs among all SNVs shared among all 4 samples (*n* = 1304), and SNVs in each sample. Asterisks indicate which base is mutated; e.g. TpC* stands for TpC- > TpA, TpC- > TpG, or TpC- > TpT. (PDF 116 kb)

## Abbreviations

APOBEC: apolipoprotein B mRNA editing enzyme catalytic polypeptide-like
LOH: loss of heterozygosity
SNV: single nucleotide variants
