# Supplementary figures and images for: Determining the origin of synchronous multifocal bladder cancer by exome sequencing
Source: BMC Cancer. 2015 Nov 9;15:871. doi: 10.1186/s12885-015-1859-8 (PMC4638097; doi:10.1186/s12885-015-1859-8)

Reference Genome

**Patient 1**

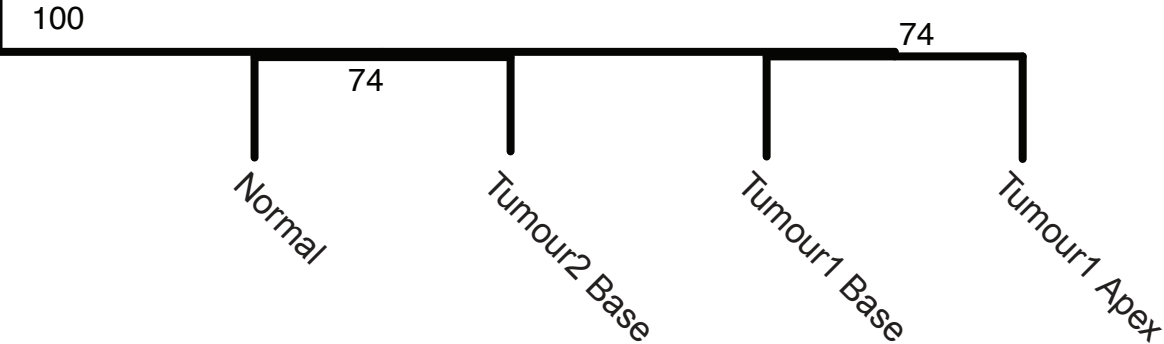

Reference Genome

**Patient 2**

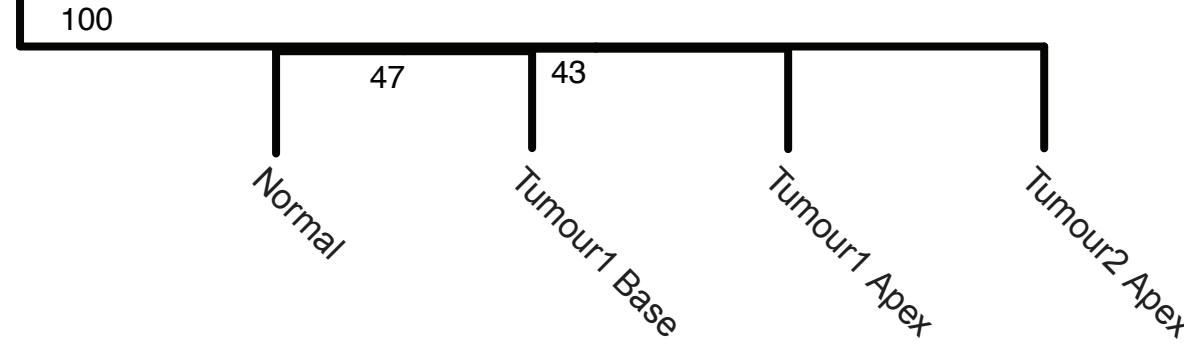

Reference Genome

**Patient 3**

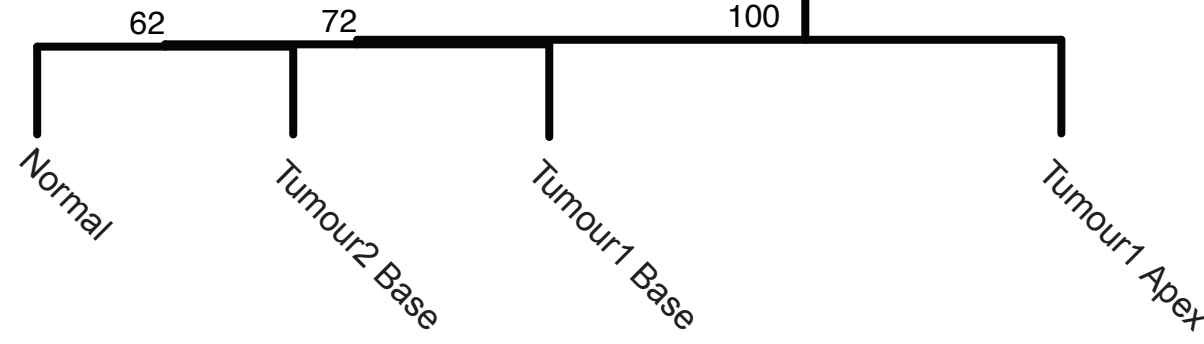

Supplement: Additional file 2: Figure S1. — Neighbour-joining tree of indels in the 4 samples and the human reference genome based on 2130–2555 indels. Bootstrap support for each internal node is indicated. (PDF 301 kb) [file 12885_2015_1859_MOESM2_ESM.pdf]

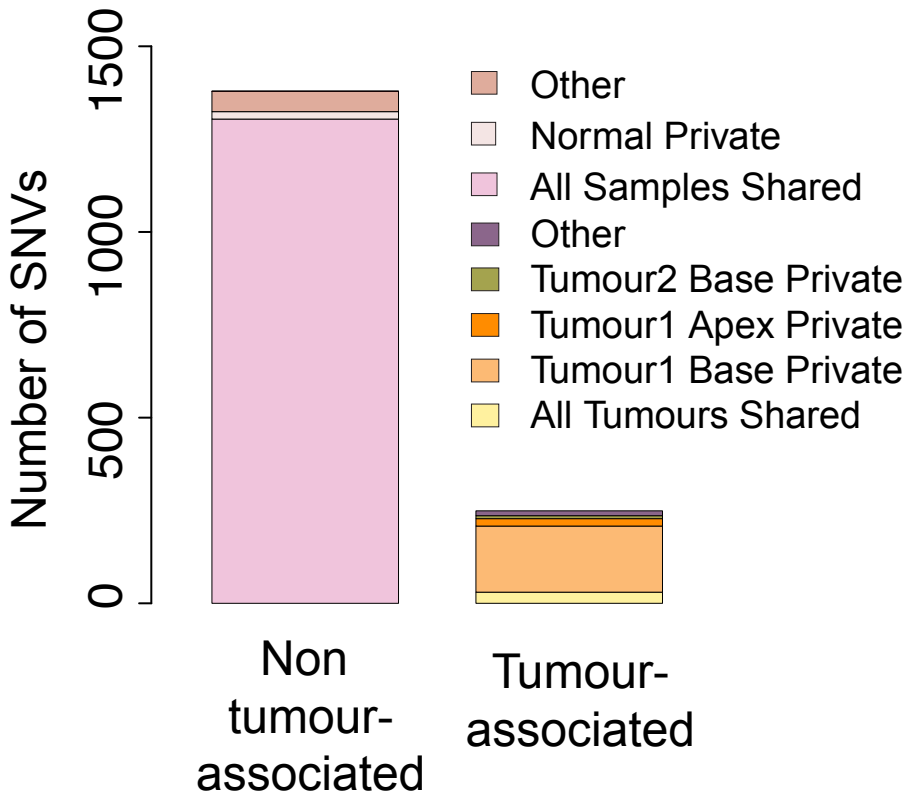

Supplement: Additional file 4: Figure S2. — Number of SNVs with respect to occurrence among samples of the Patient 3. Tumour 1 Apex, Tumour 1 Base, Tumour 2 Base, Normal private: SNVs only in that sample. All samples shared: SNVs in all 4 samples. Other: SNVs in the normal mucosa sample and in one or in two tumour samples. (PDF 140 kb) [file 12885_2015_1859_MOESM4_ESM.pdf]

Reference Genome

100

100

92

Normal

*Tumour2 Base*

*Tumour1 Apex*

*Tumour1 Base*

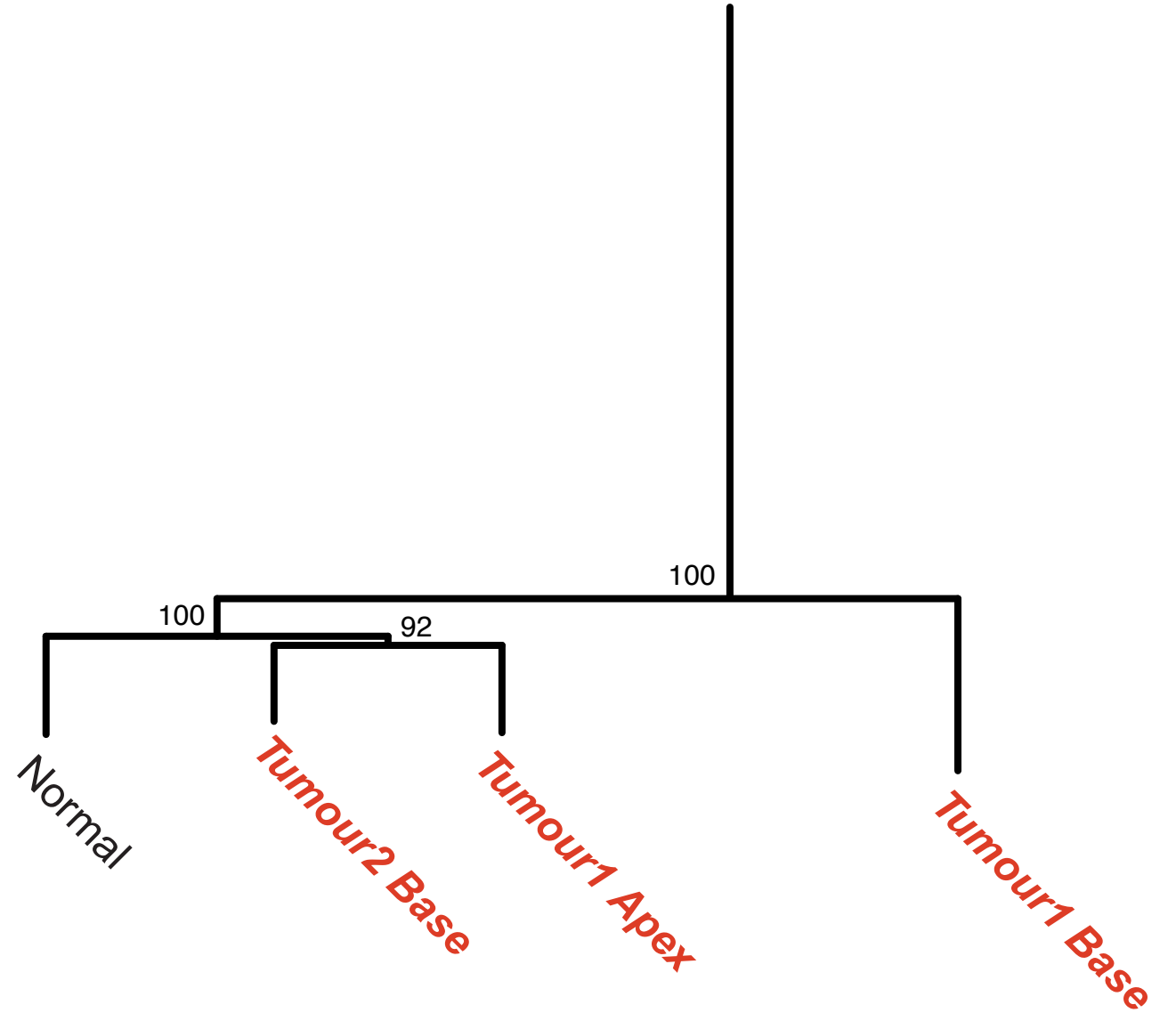

Supplement: Additional file 5: Figure S3. — Neighbour-joining tree of the 4 samples from Patient 3 and the human reference genome based on 1628 high-confidence SNVs. (PDF 119 kb) [file 12885_2015_1859_MOESM5_ESM.pdf]

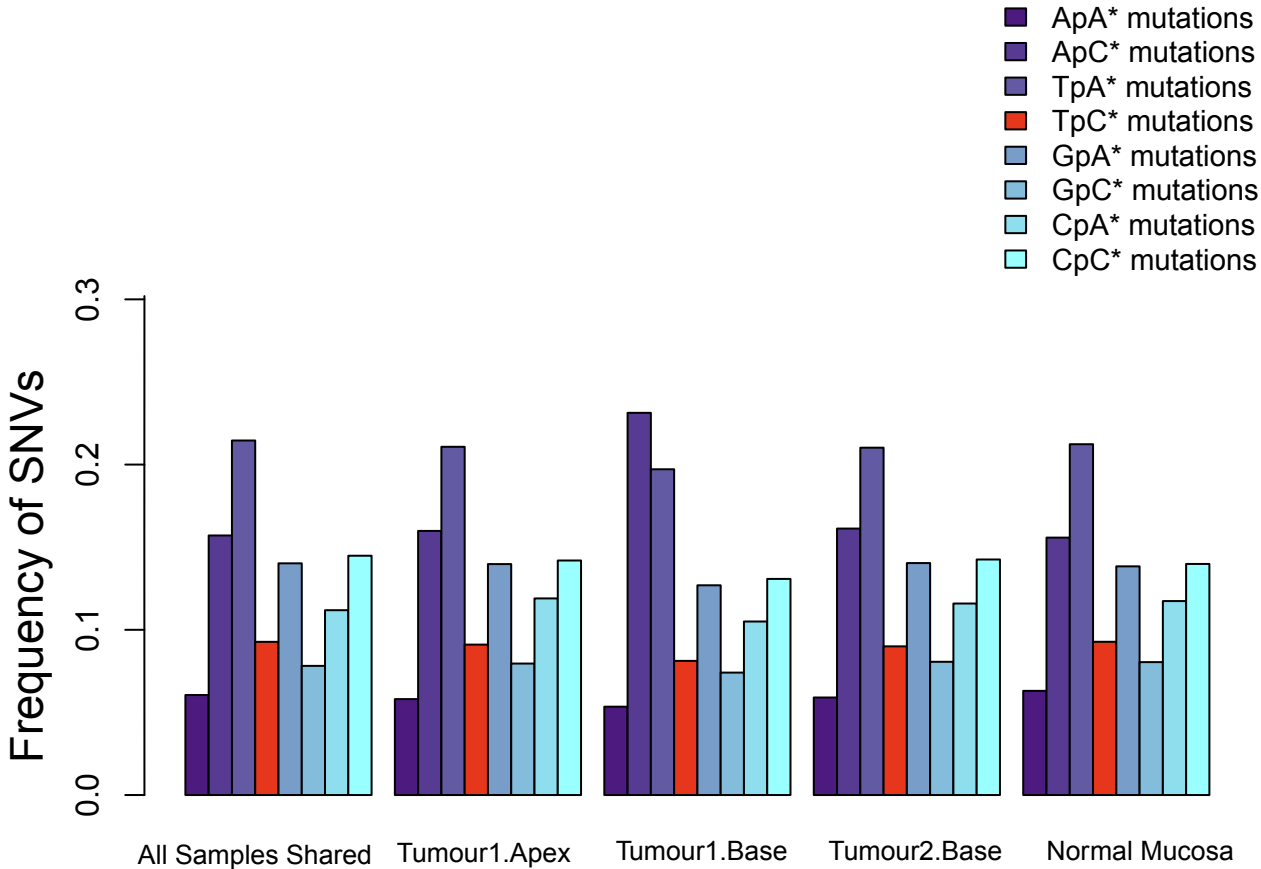

Supplement: Additional file 9: Figure S4. — Dinucleotide specific SNV frequencies in Patient 3. Frequencies are compared between SNVs among all SNVs shared among all 4 samples (n = 1304), and SNVs in each sample. Asterisks indicate which base is mutated; e.g. TpC* stands for TpC- > TpA, TpC- > TpG, or TpC- > TpT. (PDF 116 kb) [file 12885_2015_1859_MOESM9_ESM.pdf]
